# Row Ratios of Intercropping Maize and Soybean Can Affect Agronomic Efficiency of the System and Subsequent Wheat

**DOI:** 10.1371/journal.pone.0129245

**Published:** 2015-06-10

**Authors:** Yitao Zhang, Jian Liu, Jizong Zhang, Hongbin Liu, Shen Liu, Limei Zhai, Hongyuan Wang, Qiuliang Lei, Tianzhi Ren, Changbin Yin

**Affiliations:** 1 Key Laboratory of Non-point Source Pollution Control, Ministry of Agriculture, Institute of Agricultural Resources and Regional Planning, Chinese Academy of Agricultural Sciences, Beijing, 100081, PR China; 2 USDA-Agricultural Research Service, Pasture Systems and Watershed Management Research Unit, University Park, Pennsylvania, 16802, United States of America; 3 Agro-Environmental Protection Institute, Ministry of Agriculture, Tianjin, 300191, PR China; Northwest A&F University, CHINA

## Abstract

Intercropping is regarded as an important agricultural practice to improve crop production and environmental quality in the regions with intensive agricultural production, e.g., northern China. To optimize agronomic advantage of maize (*Zea mays* L.) and soybean (*Glycine max* L.) intercropping system compared to monoculture of maize, two sequential experiments were conducted. Experiment 1 was to screening the optimal cropping system in summer that had the highest yields and economic benefits, and Experiment 2 was to identify the optimum row ratio of the intercrops selected from Experiment 1. Results of Experiment 1 showed that maize intercropping with soybean (maize || soybean) was the optimal cropping system in summer. Compared to conventional monoculture of maize, maize || soybean had significant advantage in yield, economy, land utilization ratio and reducing soil nitrate nitrogen (N) accumulation, as well as better residual effect on the subsequent wheat (*Triticum aestivum* L.) crop. Experiment 2 showed that intercropping systems reduced use of N fertilizer per unit land area and increased relative biomass of intercropped maize, due to promoted photosynthetic efficiency of border rows and N utilization during symbiotic period. Intercropping advantage began to emerge at tasseling stage after N topdressing for maize. Among all treatments with different row ratios, alternating four maize rows with six soybean rows (4M:6S) had the largest land equivalent ratio (1.30), total N accumulation in crops (258 kg ha^-1^), and economic benefit (3,408 USD ha^-1^). Compared to maize monoculture, 4M:6S had significantly lower nitrate-N accumulation in soil both after harvest of maize and after harvest of the subsequent wheat, but it did not decrease yield of wheat. The most important advantage of 4M:6S was to increase biomass of intercropped maize and soybean, which further led to the increase of total N accumulation by crops as well as economic benefit. In conclusion, alternating four maize rows with six soybean rows was the optimum row ratio in maize || soybean system, though this needs to be further confirmed by pluri-annual trials.

## Introduction

Northern China has a very intensive agriculture with high inputs of seeds, irrigation and chemicals, because of high pressure of food security. This has caused severe environmental problems [1], including pollution of groundwater by nitrate from soils [2], gas emission to air [3], and soil acidification [4]. Loss of nitrogen (N) during maize (*Zea mays* L.) growth season is an especial concern, as excessive application of N is often combined with heavy summer rains in this region [5]. To ensure both food security and environmental quality, it is essential to seek best management practices, which include appropriate cropping systems that can efficiently utilize solar and soil resources with minimum nutrient inputs.

Intercropping, one type of a multiple cropping system, is recommended to be used in many parts of the world for food or fibers productions, because of its overall high productivity, effective control of pests and diseases, good ecological services and economic profitability [6–9]. In an intercropping system, there are often two or more crop species grown in the same field for a certain period of time, even though the crops are not necessarily sown or harvested simultaneously. In practice, most intercropping systems involve only two crops, as inclusion of more crops results in higher labor costs [10]. An intercropping system often consists of three phases: (1) one crop grown for a short time, (2) two intercropping crops grown simultaneously for a long time, and (3) the other crop grown for a short time [11]. The second phase is essential (or even the only phase), and it is the key phase for formation of intercropping advantage. The success of intercropping systems is due to an enhanced temporal and spatial complementarity of resource capture, for which both above-ground and below-ground parts of crops play an important role [12].

Cereal crops intercropping with legumes are a popular option in intercropping. Even though the two crops compete for soil N as they both need it for the growth, the competition drives legumes to fix atmospheric N_2_ in symbiosis with *Rhizobium* [13]. This actually results in complementary utilization of N by the crops, which is of particular importance in soils where inorganic N is limited or over-fertilized. However, negative intercropping productivity due to interspecific competition has also been reported [14], especially when the fields are managed inappropriately [11]. Therefore, only reasonable use of competitive and facilitative interactions between crops in intercropping systems can enhance crop productivity and nutrient use efficiency [15–17].

In China, intercropping is regarded to be an important agronomic practice, given the high pressure of food security due to the already large and increasing population with limited and decreasing area of arable land [15]. Among different kinds of intercropping systems, strip intercropping has the greatest advantage in terms of convenience in field management of sowing and harvest [18]. Several previous studies have reported that intercropping can increase crop yield [19], due to efficient utilization of nutrients [20] and light [21], and enhanced positive interactions between crops [22,23]. However, most of these studies were focused on effects of different intercrop species [14,24]. Rare studies have been made to investigate effects of ratio of rows between crops within a specific intercropping system. There is neither report in literature about optimum row ratio of maize intercropping with soybean (*Glycine max* L.), nor explanation of the processes behind.

In this study, we carried out two field experiments to firstly screen the optimal intercropping system of maize and legume crops, and secondly to identify the optimum ratio of rows of maize and the legume (soybean) selected in the first experiment. Specifically, we evaluated intercropping effects on crop yields, economic benefits, crop N uptake, soil nitrate-N accumulation, and its residual effects, and we investigated the reasons for advantages of intercropping systems.

## Materials and Methods

### Study area

The field experiments were conducted during 2010 to 2012 at Liucun, Xushui (38°09–39°09 N, 115°19–115°46 E), Hebei Province, North China Plain. This region has a temperate continental monsoon climate, with four distinct seasons. The site has annual mean temperature of 11.9°C, annual precipitation of 567 mm and evaporation of 1,200 mm. Annual sunshine duration is 2,745 h and the frost-free period is 184 days. Wheat (*Triticum aestivum* L.)-maize rotation is a common cropping system in this region and this system had been used during 1990–2010 on the experimental site. Each year, the field was tilled with a disk plough before sowing of wheat in October. The experimental site had a Haplic Luvisol soil (FAO classification). Physical and chemical properties of the experimental soil were determined before the start of this study (Table 1).

**Table 1:** Physical and chemical properties of the experimental soil.

Soil layer (cm)	Organic matter (g kg^-1^)	NH_4_^+^-N (mg kg^-1^)	NO_3_^-^-N (mg kg^-1^)	Bulk density (g cm^-3^)	pH
**0–20**	18.6	1.24	13.0	1.32	8.70
**20–40**	10.6	1.64	7.41	1.33	8.61
**40–60**	9.84	1.34	4.93	1.33	8.63
**60–80**	8.51	1.72	4.61	1.35	8.56
**80–100**	9.65	1.45	4.01	1.42	8.55
**100–120**	11.0	1.82	4.63	1.33	8.58
**120–140**	9.67	1.25	3.87	1.29	8.55
**140–160**	5.49	1.29	3.69	1.34	8.65
**160–180**	4.54	1.78	3.74	1.35	8.63
**180–200**	4.93	1.51	2.89	1.41	8.59

### Ethics Statement

The experimental field used in this study belongs to the Institute of Agricultural Resources and Regional Planning (IARRP) of Chinese Academy of Agricultural Sciences (CAAS), which is a national comprehensive research institution, and it has a research ethics review committee to ensure the experiment does no harm to crops, animals and humans. Our study was approved by this committee, so no specific permissions were required for the described field experiments. The sampling locations were not privately-owned or protected in any way, and this field study did not involve any endangered or protected species. In addition, there was also no vertebrate in this study.

### Experimental design

#### Experiment 1: screening of optimal intercropping system of maize and legume crops

This experiment was conducted from June 21st, 2010 to June 22nd, 2011. The experiment used a randomized complete block design, and it included five treatments with different cropping systems: (1) monoculture of maize, (2) monoculture of soybean, (3) monoculture of red bean (*Vigna angularis (Willd*.*) Ohwi et Ohashi* L.), (4) maize intercropping with soybean (Maize ǁ soybean), and (5) maize intercropping with red bean (Maize ǁ red bean). Each treatment was replicated three times. After harvest of these crops in autumn, a subsequent winter wheat crop (a 0.15 cm inter-row distance) was planted at all the plots. The experimental plot size ranged from 175 m^2^ (25 m × 7 m) to 225 m^2^ (25 m × 9 m) in different treatments, to aid practical operations in the field. Detailed planting pattern of every treatment was shown in Table 2. Maize (variety Zhengdan 958, Henan Academy of Agricultural Sciences), soybean (variety Zhonghuang 30, Chinese Academy of Agricultural Sciences) and red bean (variety Jinhong 1, Heishan Jinyu Seed Co. Limited of Liaoning province) were all sown on June 21, 2010. The crops were irrigated after sowing, thinned out after crop emergence, and weeded in time during crop growth. Red bean was harvested on September 13th, and maize and soybeans were harvested on October 6. Wheat (variety Tangmai 6, Tangshan Academy of Agricultural Sciences) was sown on October 8, 2010, and harvested on June 22, 2011.

**Table 2:** Detailed planting pattern of every treatment in the two experiments.

Experiment/Crop		Row spacing (cm)^♣^	Plant spacing (cm)	Width of strip (cm)	Spacing between two crop strips (cm)	Plot area (m^2^)	Crop density (plant ha^-1^)
**Experiment 1**							
Maize monoculture		80, 50	25	-	-	175	60,984
Soybean monoculture		35	20	-	-	175	283,216
Red bean monoculture		30	20	-	-	175	330,584
Maize ǁ soybean	Maize	80, 50	25	180	20	225	52,866
	Soybean	27	20	100			263,872
Maize ǁ red bean	Maize	80, 50	25	180	20	225	52,866
	Red bean	27	20	100			263,872
**Experiment 2**							
Maize monoculture		80, 50	25	-	-	70	60,984
Soybean monoculture		35	20	-	-	70	283,216
2M:6S	Maize	50	25	50	30	110	30,300
	Soybean	30	20	180			229,200
4M:6S	Maize	50	25	150	30	110	44,000
	Soybean	30	20	180			165,200
6M:6S	Maize	50	25	250	30	110	51,100
	Soybean	30	20	180			129,500

^♣^ Maize was sown with alternating wide (80 cm) and narrow (50 cm) inter-row spacing in Experiment 1 and maize monoculture in Experiment 2, while intercropped maize in Experiment 2 was sown with equal row spacing (50 cm).

#### Experiment 2: identification of optimum ratio of rows of maize and soybean

**Fig 1:**
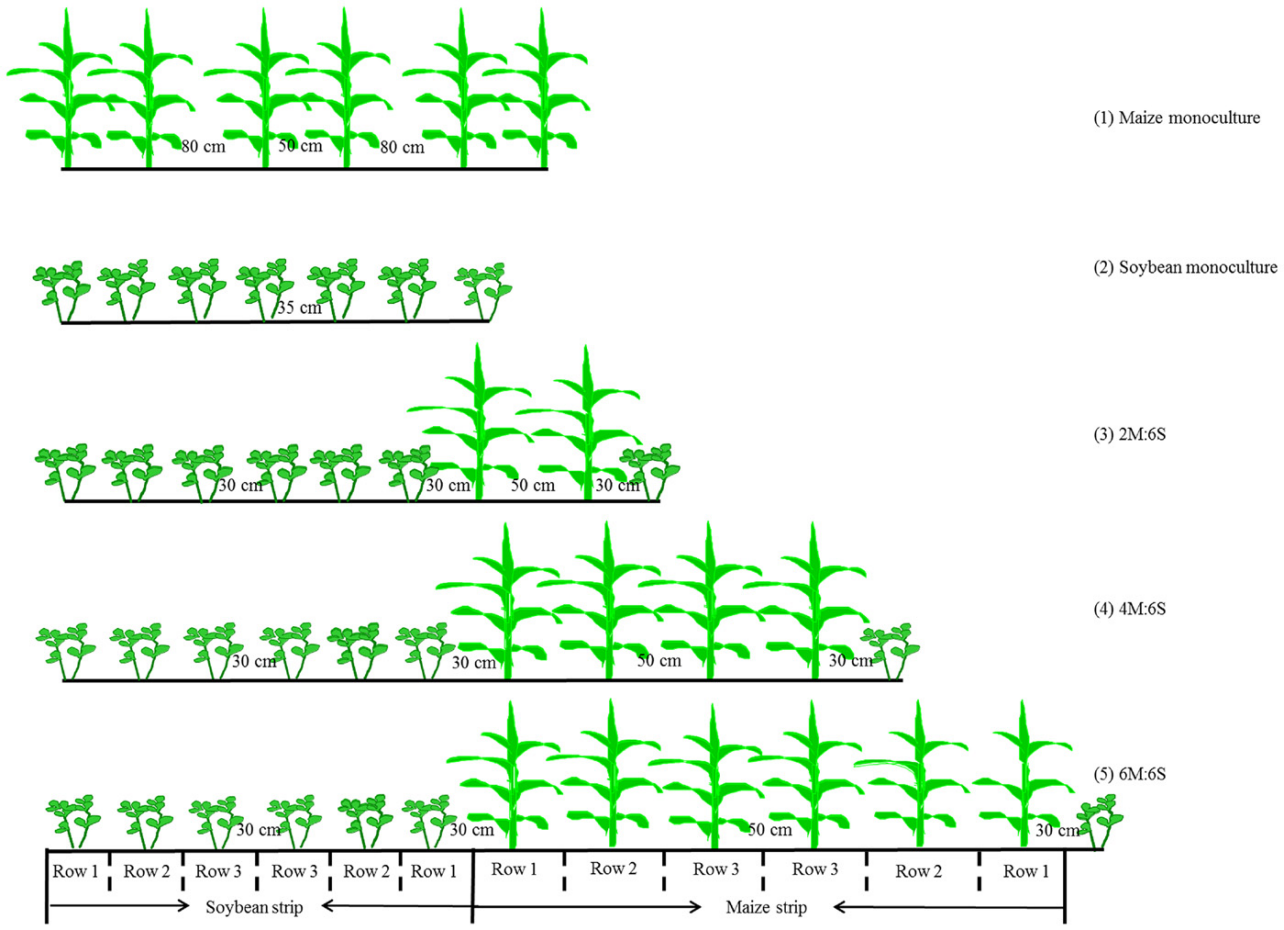
Schematic diagram of different treatments. (1) Monoculture of maize, (2) Monoculture of soybean, (3) 2M:6S (two maize rows intercropping with six soybean rows), (4) 4M:6S (four maize rows intercropping with six soybean rows), and (5) 6M:6S (six maize rows intercropping with six soybean rows).

Experiment 2 was conducted to identify optimum ratio of rows of maize and soybean, as maize ǁ soybean was identified as the best intercropping system in Experiment 1. A randomized complete block design, which included five treatments with different planting patterns in three replicates, was used in summer, 2011. The treatments were: (i) monoculture of maize, (ii) monoculture of soybean, (iii) alternating two maize rows with six soybean rows (2M:6S), (iv) alternating four maize rows with six soybean rows (4M:6S), and (v) alternating six maize rows with six soybean rows (6M:6S) (Fig 1). Detailed planting pattern of every treatment was shown in Table 2. Maize and soybean were sown on June 24^th^, 2011, and harvested on October 6^th^, 2011. A subsequent winter wheat crop was sown on October 7^th^, 2011 with a 15 cm inter-row spacing, and it was harvested on June 17^th^, 2012.

In both experiments, maize, winter wheat, soybean and red bean were supplied with 225, 225, 45 and 45 kg N ha^-1^ in urea (46% N), respectively, and all crops were supplied with 33 kg P ha^-1^ in calcium superphosphate (12% P) and 62 kg K ha^-1^ in potassium sulphate (52% K). For maize and wheat, half of the N was incorporated into the top 20 cm soil as base fertilizers at sowing, and the rest half was applied during the jointing stage, which was 40 days after planting (DAP). All N for beans, and all P and K fertilizers for all crops were applied as basal fertilizers. Five flooding irrigations were applied to wheat, with one irrigation of 50 mm water at wheat growth stages of sowing, overwinter, erecting, booting and filling, respectively.

### Sample collection and measurement

Before the start of the study in June 2010, soil was sampled randomly in the field by using a soil auger for every 20 cm soil depth until 200 cm deep, to determine basic physical and chemical properties of each soil layer. In Experiment 1, soil samples were collected from every crop strip with the same procedure as described above, both after harvest of summer crops and after harvest of winter wheat. In Experiment 2, soil samples (0–20 cm and 20–40 cm) were collected in inter-row (Fig 1) during maize growth season, at maize pre-jointing stage (BBCH19, 26 DAP, the jointing stage was 40 days after planting), tasseling stage (BBCH51, 57 DAP), filling stage (BBCH73, 74 DAP) and ripe stage (BBCH89, 104 DAP), respectively. After harvest of wheat in 2012, soil samples were collected from each plot for every 20 cm depth until 100 cm deep, and the samples were taken separately under the previously established summer maize and soybean strips.

Soil water content was determined by oven-drying 20 grams of fresh soil at 105°C for 12 h to a constant mass. Soil mineral N (NH_4_^+^-N and NO_3_^-^-N) concentrations were determined with a continuous flow analyzer (TRAACS 2000; Bran and Luebbe, Norderstedt, Germany), after extracting 12 grams of fresh soil with 100 ml 0.01 M CaCl_2_ [20]. Soil organic matter was determined by the potassium dichromate method, total N by the automatic Kjeldahl method after wet digestion [25], and soil water pH by using a standard calomel electrode [26].

In Experiment 1, five plants of maize or bean were sampled from each crop strip after harvest in summer. In Experiment 2, plant samples of maize and soybean were collected to determine dry matter content at maize pre-jointing stage (26 DAP), tasseling stage (57 DAP) and ripe stage (104 DAP), respectively. At each stage, 10 plants of each crop were sampled from every row (Fig 1). Wheat plant samples within an area of 2 m^2^ were taken separately for the previously established maize or soybean strip. Stalks and grains of the crops were harvested separately at ripe stage. All the samples were oven-dried at 105°C for 30 min and then at 85°C until constant weights. Thereafter, dry plant samples were ground to determine total N concentrations by the automatic Kjeldahl method, after wet digestion of the samples with H_2_SO_4_ and H_2_O_2_ [27].

Photosynthetic characteristics of maize were measured in each row using an infrared gas analyzer-based photosynthesis system (LI-6400, Li-Cor., Lincoln, NE, USA) at tasseling stage (57 DAP) and filling stage (74 DAP). The measurements took place at the middle of the ear leaf during normal weather conditions with sunshine and no rain in summer, between 9:00–12:00 in the morning. Based on the literatures [28–30] and the actual circumstances, the conditions supported were as follows: photosynthetic photon flux density of 1800 μmol m^-2^ s^-1^, CO_2_ concentration of 400 μmol mol^-1^, air temperature of 30°C and relative air humidity of 0% (0% moisture from air to the leaf chamber).

### Calculation and Statistical analysis

Land equivalent ratio (LER), which is often considered as an indicator of intercropping benefit [31], was calculated according to:

$$\text{LER}=\frac{Y_{im}}{Y_{sm}}+\frac{Y_{is}}{Y_{ss}}$$ (1)

where *Y*_*im*_ (kg ha^-1^) and *Y*_*is*_ (kg ha^-1^) are respective yields of intercropped maize and soybean or red bean per ha intercropping area, and *Y*_*sm*_ (kg ha^-1^) and *Y*_*ss*_ (kg ha^-1^) are yields of maize and bean in monoculture treatments. If LER is greater than 1.00, there is a yield advantage by intercropping; otherwise there is no yield advantage.

Total N accumulated by the crop (Nacc, kg ha^-1^) was calculated according to:

Nacc=∑[M×C] (2)

where *M* is the amount of dry matter (kg ha^-1^) and *C* is the N concentration in the plant (%).

Economic benefit (E, USD ha^-1^) was calculated according to:

E=Y×P−LF−FF−SF−MF (3)

where *Y* is yield (kg ha^-1^), *P* is grain price (USD ha^-1^), *LF* is labor fees (USD ha^-1^), *FF* is fertilizer fees (USD ha^-1^), *SF* is seed costs (USD ha^-1^) and *MF* is machinery expenses (USD ha^-1^). USD is U.S. dollar.

Nitrate-N accumulation in soil (R, kg ha^-1^) was calculated according to:

$$\text{R}=\frac{T\times B\times C}{10}$$ (4)

where *T* is the thickness (cm) of a soil layer, *B* is soil bulk density (g cm^-3^), and *C* is concentration of soil nitrate-N (mg kg^-1^).

Analysis of variance (ANOVA) was conducted using the SPSS19.0 software package and mean values (*n* = 3) were compared by least significant difference at the 5% level.

## Results

### Screening of optimal intercropping system of maize with legume

Compared to monoculture, both maizeǁsoybean and maizeǁred bean cropping systems had intercropping advantages in yield and economy (i.e., promoted crop yields and farmers’ income) (Table 3). Both intercropping systems had a LER value greater than 1. Maizeǁsoybean had the highest economic benefit among all the cropping systems, but it did not significantly differ with that of maizeǁred bean. In addition, both maizeǁsoybean and maizeǁred bean cropping systems significantly reduced soil (0–200 cm) nitrate-N accumulation compared to monoculture of maize (*P*<0.05). Maizeǁsoybean had soil nitrate-N accumulation 56 kg ha^-1^ lower than monoculture of maize, and 18 kg ha^-1^ lower than maizeǁred bean. In addition, compared to monoculture of maize, both maizeǁsoybean and maizeǁred bean did not significantly affect yield of subsequent wheat, but they reduced nitrate-N accumulation in soil (0–200 cm) after harvest of subsequent wheat (Table 4). Considering all the aspects above, maizeǁsoybean performed better than maizeǁred bean and all monocultures, and thus maizeǁsoybean was investigated with further details.

**Table 3:** Crop yield, economic benefit, soil nitrate-N accumulation at 0–200 cm soil depth (NA) and land equivalent ratio (LER) in different crop systems at harvest in the summer of 2010 (Experiment 1).

Treatments		Yield (kg ha^-1^)	LER	Economy^¶^ (USD ha^-1^)	NA (0–200cm) (kg ha^-1^)
**Maize monoculture**		11,003	-	2,437 a	192 a
**Soybean monoculture**		3,091	-	1,367 b	95 c
**Red bean monoculture**		2,583	-	1,203 b	107 c
**Maizeǁsoybean**	**Maize**	9,142	1.27	2,755 a	136 bc
	**Soybean**	1,345			
**Maizeǁred bean**	**Maize**	9,139	1.30	2,747 a	154 b
	**Red bean**	1,203			

Data were presented on a basis of per hectare monoculture/intercropping area

^¶^Cost: Machinery 46.44 USD ha^-1^, labor 216.7 USD ha^-1^, Maize seed 1.24 USD kg^-1^, soybean seed 1.86 USD kg^-1^, red bean seed 3.10 USD t^-1^, urea 325.08 USD t^-1^, calcium superphosphate 123.8 USD t^-1^, potassium sulfate 541.8 USD t^-1^; Procurement price: maize grain 0.28 USD kg^-1^, maize straw 0.003 USD kg^-1^, soybean grain 0.62 USD kg^-1^, red bean grain 0.70 USD kg^-1^.

Note: Different letters represented significant differences between means of replicates (n = 3) at the 0.05% level within a column.

**Table 4:** Residual effect of different summer cropping systems on yield of winter wheat and soil nitrate-N accumulation after harvest of wheat in Experiment 1.

Treatments in summer	Yield of wheat (kg ha^-1^)	Soil nitrate-N accumulation (0–200cm) (kg ha^-1^)
**Maize monoculture**	7,633 a	260 a
**Soybean monoculture**	7,633 a	204 c
**Red bean monoculture**	7,633 a	201 c
**Maizeǁsoybean**	7,830 a	233 b
**Maizeǁred bean**	7,695 a	229 b

Note: Different letters represented significant differences between means of replicates (n = 3) at the 0.05% level within a column.

### Effects of row ratios on agronomy and environmental benefits of intercropping systems

#### Overall effects of row ratios

Despite being applied with less N, intercropping significantly increased crop yields, economic benefit and crop total N uptake (*P*<0.05), and reduced nitrate-N accumulation in soil at harvest, compared to crop monocultures on equivalent land areas. Specifically, intercropping increased maize yield (per hectare maize growing area) by 42.2–92.3%. Soybean yield was slightly decreased by 6.5% in 2M:6S and by 4.4% in 6M:6S, but it was increased by 3.0% in 4M:6S. As a result, the LER values of the three intercropping systems were all greater than 1, and the land utilization rates in these systems were 24–30% higher than the rates in the two monocultures (Table 5). 4M:6S had the best economic benefit, which was 26.0% higher than maize monoculture, 45.4% higher than soybean monoculture, 8% higher than 2M:6S, and 5% higher than 6M:6S. Amounts of total N accumulation in crops were similar in 4M:6S (258 kg ha^-1^) and 2M:6S (257 kg ha^-1^), which were significantly higher than those in the other treatments (*P*<0.05). Soil nitrate-N accumulation (0–40 cm) at harvest was smaller at lower proportion of maize growing area. Compared to maize monoculture, intercropping significantly reduced soil N accumulation after crop harvest, by 46.6% in 6M:6S, 57.6% in 4M:6S and 65.1% in 2M:6S.

**Table 5:** Total N accumulation in crop (Nacc), crop yield, economy, soil nitrate-N accumulation at 0–40 cm soil depth (NA) and land equivalent ratio (LER) in different cropping systems at harvest in the summer of 2011 (Experiment 2).

Cropping system	Maize				Soybean				LER
	Nacc (kg ha^-1^)	Yield (kg ha^-1^)	Economy^¶^ (USD ha^-1^)	NA (kg ha^-1^)	Nacc (kg ha^-1^)	Yield (kg ha^-1^)	Economy^¶^ (USD ha^-1^)	NA (kg ha^-1^)	
**Maize monoculture**	160 a	9,630 a	2,827 a	63.3 a	-	-	-	-	-
**Soybean monoculture**	-	-	-	-	237 a	3,780 a	2,457 a	22.8 a	-
**2M:6S**	93 d	5,700 d	1,762 d	6.9 d	164 b	2,440 b	1,534 b	23.2 a	1.24
**4M:6S**	129 c	7,610 c	2,330 c	20.0 c	129 c	1,950 c	1,234 c	16.5 b	1.30
**6M:6S**	139 b	8,340 b	2,533 b	32.3 b	96 d	1,410 d	872 d	13.7 b	1.24

Data were presented on a basis of per hectare monoculture/intercropping area.

^¶^Cost: Machinery 46.44 USD ha^-1^, labor 216.7 USD ha^-1^, Maize seed 1.86 USD kg^-1^, soybean seed 1.86 USD kg^-1^, urea 371.5 USD t^-1^, calcium superphosphate 123.8 USD t^-1^, potassium sulfate 541.8 USD t^-1^; Procurement price: maize grain 0.34 USD kg^-1^, maize straw 0.003 USD kg^-1^, soybean grain 0.77 USD kg^-1^.

Note: Different letters represented significant differences between means of replicates (n = 3) at the 0.05% level within a column.

#### Photosynthetic characteristics of maize at different growth stages

Intercropping enabled an efficient utilization of environmental resources. At maize tasseling and filling stages, photosynthetic gas-exchange parameters, including net photosynthetic rate (P_n_) and transpiration rate (T_g_), collectively showed a border row effect [32]. That is, values of photosynthetic characteristics were larger at a smaller distance to the border row within a maize strip (Table 6). At both stages, the P_n_ of maize in row 1 of 4M:6S and 6M:6S were significantly (*P*<0.05) higher than that in the inner-row in these treatments as well as that in maize monoculture. The T_g_ of maize in row 1 did not significantly differ between intercropping systems, but they were all significantly (*P*<0.05) higher than that in maize monoculture. Moreover, T_g_ of maize in the inner-row in intercropping systems was also slightly higher than that in maize monoculture.

**Table 6:** Net photosynthetic rate (P_n_) and transpiration rate (T_g_) of maize at tasseling and filling stages under different cropping systems.

Growth stage/Cropping system	P_n_ (mmol m^-2^ s^-1^)			T_g_ (mol m^-2^ s^-1^)		
	Row 1	Row 2	Row 3	Row 1	Row 2	Row 3
**Tasseling**						
Maize monoculture	39.2 c	39.2 c	39.2 c	9.17 de	9.17 de	9.17 de
2M:6S	40.2 bc	-	-	11.2 ab	-	-
4M:6S	42.8 a	39.3 c	-	11.5 a	9.94 cd	-
6M:6S	41.5 ab	39.7 c	39.0 c	11.9 a	10.5 bc	8.69 e
**Filling**						
Maize monoculture	38.5 c	38.5 c	38.5 c	6.56 b	6.56 b	6.56 b
2M:6S	39.2 bc	-	-	7.06 a	-	-
4M:6S	40.6 ab	38.7 c	-	7.06 a	6.66 ab	-
6M:6S	41.2 a	38.7 c	38.2 c	7.08 a	6.70 ab	6.42 b

Note: Different letters represented significant (at the 0.05% level) differences between means of three replicates among all crop rows of different treatments at each growth stage.

#### Uptake of N by crops at different growth stages

Intercropping systems did not reduce N concentrations in maize or soybean compared to monocultures (Table 7). At each growth stage, N concentration of maize in all intercropping systems did not significantly differ from that of maize monoculture (*P*<0.05), except that N concentration of maize in 2M:6S was significantly higher than that in maize monoculture at pre-jointing stage. The N concentration of soybean monoculture was significantly lower than that of the intercropped soybean (*P*<0.05) at the early growth stage of pre-jointing, but they did not differ at tasseling and ripening stages. At pre-jointing stage, the N concentration of the intercropped soybean descended from row 1 to row 3, while the concentration did not significantly differ between rows at tasseling and ripening stages. At ripening stage, the soybean straw had much lower N concentration (3.64–4.77 mg kg^-1^) than the grains (60.71–66.54 mg kg^-1^). For the entire growth period, intercropping increased total N accumulation of the whole system compared to monocultures (Table 5).

**Table 7:** Concentration (g kg^-1^) of N in maize and soybean at each growth stage under different cropping systems.

Growth stage/Cropping system	Maize strip			Soybean strip		
	Row 1	Row 2	Row 3	Row 1	Row 2	Row 3
**Pre-jointing**						
Monoculture	29.5 b	29.5 b	29.5 b	29.7 e	29.7 e	29.7 e
2M:6S	32.9 a	-	-	38.1 b	40.9 a	37.8 b
4M:6S	28.4 b	31.1 ab	-	37.6 b	28.8 e	34.7 c
6M:6S	29.9 ab	29.9 ab	30.42 ab	38.3 b	35.2 c	32.6 d
**Tasseling**						
Monoculture	17.0 a	17.0 a	17.0 a	27.5 a	27.5 a	27.5 a
2M:6S	17.0 a	-	-	27.2 a	27.5 a	26.5 a
4M:6S	16.8 a	15.0 a	-	26.9 a	25.6 a	26.9 a
6M:6S	14.8 a	16.3 a	15.5 a	27.2 a	28.0 a	27.1 a
**Ripening-straw**						
Monoculture	7.78 ab	7.78 ab	7.78 ab	3.73 de	3.73 de	3.73 de
2M:6S	7.39 ab	-	-	4.25 ab	4.77 a	3.89 cde
4M:6S	8.10 a	7.19 b	-	3.64 e	3.92 bcd	4.66 ab
6M:6S	7.56 ab	8.13 a	7.58 ab	4.22 ab	4.25 ab	4.42 ab
**Ripening-grain**						
Monoculture	11.7 a	11.7 a	11.7 a	60.7 b	60.7 b	60.7 b
2M:6S	11.6 a	-	-	64.4 ab	65.5 ab	65.0 ab
4M:6S	12.5 a	12.5 a	-	63.2 ab	62.3 ab	64.5 ab
6M:6S	11.7 a	11.8 a	12.2 a	66.5 a	65.7 ab	65.3 ab

Note: Different letters represented significant (at the 0.05% level) differences between means of three replicates among all crop rows of different treatments in one crop strip at each growth stage.

#### Crop biomass at different growth stages

Crop biomass per plant gradually increased from seeding to ripening stage (Table 8). At pre-jointing, biomass of intercropped maize and soybean (except maize row 1) in 2M:6S was significantly lower than biomass of monoculture (*P*<0.05). 2M:6S and 6M:6S showed a negative border row effect on biomass per plant, but the effect was positive in 4M:6S. Positive border row effect on maize biomass started to appear at tasseling stage (Table 8), when biomass per soybean plant in intercropping showed an advantage of inner rows and gradually increased from row 1 to row 3.

**Table 8:** Crop biomass (g plant^-1^) at each growth stage under different cropping systems.

Growth stage/Cropping system	Maize strip			Soybean strip		
	Row 1	Row 2	Row 3	Row 1	Row 2	Row 3
**Pre-jointing**						
Monoculture	6.79 a	6.79 a	6.79 a	2.70 a	2.70 a	2.70 a
2M:6S	6.89 a	-	-	1.75 d	2.14 bc	2.38 b
4M:6S	5.47 c	5.43 c	-	2.37 b	1.97 cd	1.76 d
6M:6S	5.85 b	4.92 d	5.49 c	1.96 cd	2.04 cd	2.14 bc
**Tasseling**						
Monoculture	90.0 bc	90.0 bc	90.0 bc	17.5 a	17.5 a	17.5 a
2M:6S	104 a	-	-	11.9 c	13.8 bc	16.3 a
4M:6S	95.4 ab	81.7 c	-	11.9 c	15.6 ab	16.3 a
6M:6S	96.3 ab	94.8 ab	92.4 b	11.9 c	12.5 c	15.6 ab
**Ripening**						
Monoculture	257 b	257 b	257 b	20.6 b	20.6 b	20.6 b
2M:6S	311 a	-	-	15.3 de	16.0 de	17.7 c
4M:6S	299 a	259 b	-	16.7 cd	20.5 b	22.7 a
6M:6S	289 a	252 b	248 b	15.0 e	16.4 cde	16.6 cd

Note: Different letters represented significant (at the 0.05% level) differences between means of three replicates among all crop rows of different treatments in one crop strip at each growth stage.

At ripening stage, row 1 of all intercropped maize had an obvious advantage and its biomass per plant was significantly higher than that of monoculture of maize (*P*<0.05), but this was not observed for the maize inner rows (row 2 and row 3). The biomass per intercropped soybean plant gradually increased from row 1 to row 3, but all biomass except in 4M:6S were significantly lower than that of soybean monoculture (*P*<0.05). Overall, 4M:6S had an advantage in row 2 and row 3 of the intercropped soybean compared with monoculture, but there was always a disadvantage in 2M:6S and 6M:6S.

#### Soil nitrate-N concentration at different growth stages

**Fig 2:**
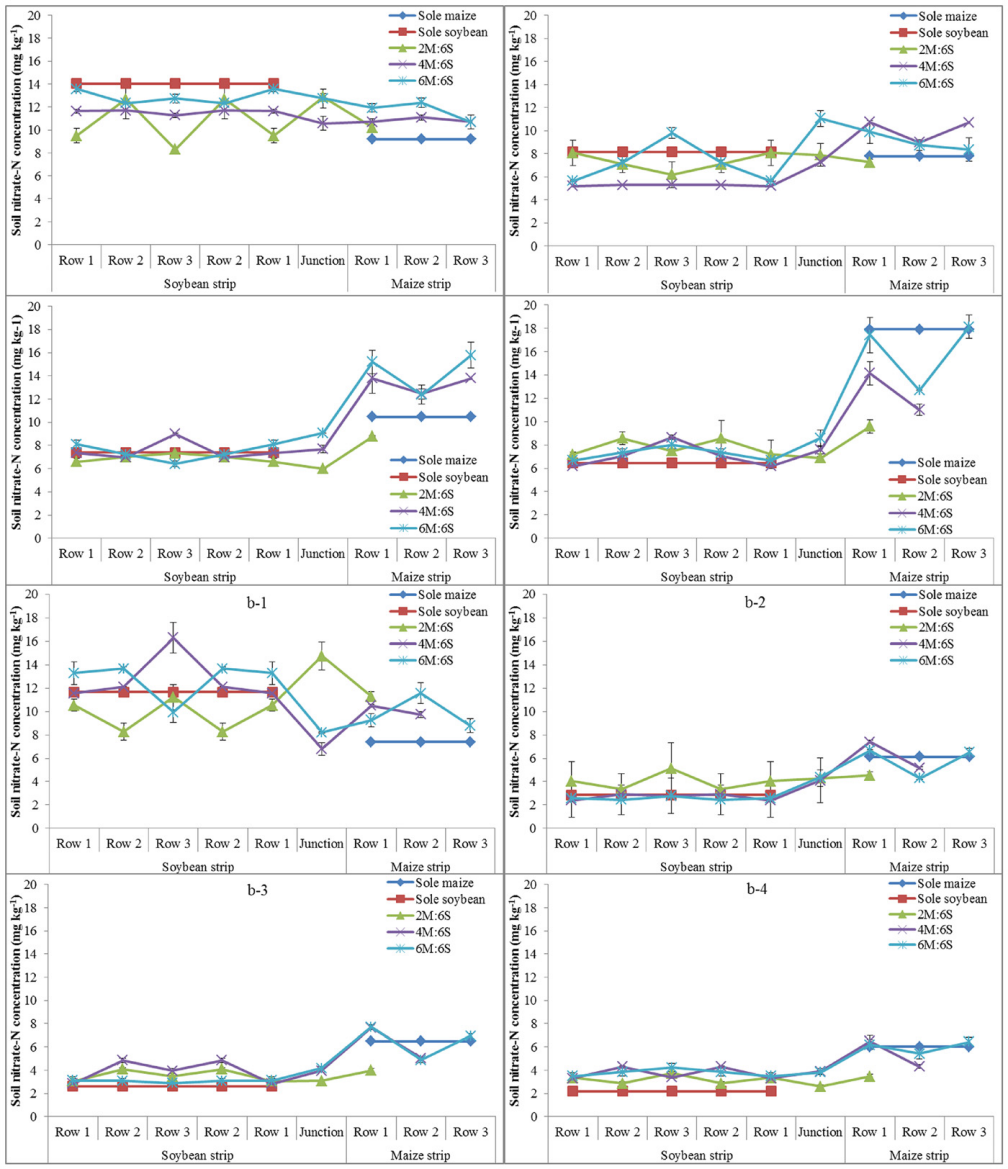
Spatial distribution of nitrate-N concentration (mg kg^-1^) of each cropping system at different soil depths and maize growth stages. (a) a soil depth of 0–20 cm: (a-1) pre-jointing stage, (a-2) tasseling stage, (a-3) filling stage and (a-4) ripening stage, and (b) a soil depth of 20–40 cm: (b-1) pre-jointing stage, (b-2) tasseling stage, (b-3) filling stage and (b-4) ripening stage. A row number is defined as in Fig 1. Each data point presents mean of three replicates and an error bar indicated standard deviation.

Nitrate-N concentration in top-soil (0–20 cm) was obviously higher than that in sub-soil (20–40 cm). In the soybean strip, nitrate-N concentration in both soil layers gradually decreased from pre-jointing to ripening stage of soybean, while for maize the concentration decreased from pre-jointing to tasseling stage and increased after topdressing N until ripening stage (Fig 2). Before N topdressing for maize, soil nitrate-N concentration (0–20 cm and 20–40 cm) in rows of maize monoculture was significantly lower than that of all intercropping rows. After N topdressing, nitrate-N concentration at 0–20 cm soil depth of all maize rows gradually increased until harvest. In particular, the nitrate-N concentration in maize monoculture had a dramatic amplification until reaching the highest point at ripening stage. However, N topdressing did not substantially affect soil N concentration at the depth of 20–40 cm (6.03–6.47 mg kg^-1^). Soil nitrate-N concentration (0–20 cm and 20–40 cm) in rows of soybean monoculture gradually decreased until reaching the lowest point at ripening stage compared to intercropped soybean. Except at pre-jointing stage, soil nitrate-N concentration in all soybean rows had a small variation, especially at filling stage (6.41–8.98 mg kg^-1^ in 0–20 cm and 2.62–4.84 mg kg^-1^ in 20–40 cm) and ripening stage (6.18–8.65 mg kg^-1^ in 0–20 cm and 2.19–4.30 mg kg^-1^ in 20–40 cm). Soil nitrate-N concentration in the junction row (between maize strip and soybean strip) also differed with intercropping systems, but with no clear trends. In the later period of crop growth especially after filling stage of maize, soil nitrate-N concentration in junction rows was higher than that of soybean row 1, but lower than that of maize row 1.

#### Yield of subsequent wheat and soil nitrate-N accumulation

Intercropping in summer did not significantly affect yield of winter wheat, but it significantly reduced soil nitrate-N accumulation (0–100 cm) after harvest of wheat, compared to monoculture of maize in summer (Table 9). 4M:6S produced 12–263 kg ha^-1^ higher wheat yields than the other four cropping systems, which indicated that intercropping systems in summer could even slightly increase yield of the subsequent crop. Compared to maize monoculture, intercropping systems reduced soil nitrate-N accumulation during wheat season by 21.9–51.7%.

**Table 9:** Residual effect of different summer cropping systems on yield of winter wheat and soil nitrate-N accumulation (0–100 cm) after harvest of wheat in Experiment 2.

Cropping system in summer	Subsequent crop	Yield (kg ha^-1^)	Soil nitrate-N accumulation (kg ha^-1^)
**Maize monoculture**	wheat	7,380 a	206 a
**Soybean monoculture**	wheat	7,190 a	125 b
**2M:6S**	wheat	7,150 a	97 c
**4M:6S**	wheat	7,410 a	119 bc
**6M:6S**	wheat	7,180 a	161 ab

Note: Different letters represented significant differences between means of replicates (n = 3) at the 0.05% level within a column.

## Discussion

This study clearly demonstrated that intercropping systems presented advantage over maize monoculture. Intercropping system of maize with legumes reduced N application in the same planting area compared to maize monoculture, probably because of the enhanced biological N fixation by legumes [6]. Both maizeǁsoybean and maizeǁred bean systems showed intercropping advantages in yield, economy, land utilization ratio and reducing soil nitrate-N accumulation, as well as better residual effect on the subsequent wheat crop. Previous studies had also reported beneficial effects of intercropping systems on yield, economy and the environment [6,33], which stresses the importance of using intercropping in sustainable agriculture to alleviate pressure in intensive farming systems with high inputs and outputs [14]. Soybean is more important than red bean in China, with more consumption and relying on import [34], and decreasing planting area year by year [35]. Therefore, considering maize intercropping with soybean as the best cropping system in summer in the present study is reasonable and necessary.

In particular, the optimal intercropping system was strip intercropping of 4 maize rows with 6 soybean rows (4M:6S), which had positive effects on yield, economy and environment in this study. Its LER value, crop N uptake, yields of both maize and soybean and economic benefit were even greater than those of 2M:6S and 6M:6S. This confirmed the previous finding that row ratio can influence intercropping efficiency [36,37]. In addition, 4M:6S significantly reduced soil nitrate-N accumulation compared to maize monoculture after harvest of summer crops. The LER values greater than 1 in all intercropping systems in the present study indicated high land-use efficiency compared to monoculture of maize or soybean [36].

Advantage of intercropping is probably derived from high light use efficiency above-ground and nutrients (e.g., N) below-ground [22]. Ability of maize to capture sunlight was enhanced at border rows, while there was small difference in photosynthetic rate and transpiration rate between inner-rows within a strip. The best light use efficiency was obtained in 4M:6S with narrow strips and a high proportion of border rows. Enhanced photosynthesis existed only in the two side rows, which indicated that four maize rows consisted of the optimal maize strip for light utilization. As a result, maize yield of intercropping systems was linearly correlated with photosynthetic efficiency, and light transmission was affected by between-row spacing, which supported previous findings by Prasad and Brook [38]. Intercropped soybean probably facilitated growth of maize by transferring the N fixed [39]. However, more N fertilization would inhibit N fixation of legumes [40], thus N was applied as basal fertilizer to both maize and soybean but only topdressed for maize in this study. Results showed that intercropping could provide enough nitrate for crops during the whole growth period. Top-soil was the main source of nitrate for crops, with significantly higher nitrate concentration than that of subsoil. Soil nitrate had different patterns in different intercropping systems from seeding to ripening stage. Soil nitrate-N in maize monoculture gradually increased to the highest value at ripening stage compared to intercropped maize, while soil nitrate-N in soybean monoculture gradually decreased to the lowest value at ripening stage compared to intercropped soybean. Elsewhere, Ossom et al. (2009) also observed significant differences in soil nitrate-N between different intercropping systems. Our results showed that soil nitrate-N accumulation increased gradually with the increasing maize area, probably partly because the N fertilizer rate for per unit area of maize was higher than that for soybean after topdressing.

Intercropping advantage was most obvious in 4M:6S, but the advantage did not emerge at the beginning of growth. At the early stage, biomass per plant of most intercropped crops was smaller than the corresponding monoculture, probably because maize suffered border row effect and soybean growth was also negatively affected [32]. In the middle stage with two intercropping crops grown simultaneously, different cropping systems probably had different extents of interactions between interspecific competition and facilitation [13], which led to different growth rhythm. Intercropping advantage of maize started to emerge at tasseling and lasted until ripening stage, by showing a border row effect. In contrast, intercropping advantage did not appear in soybean during the entire growth period, except in 4M:6S which had a slightly increased soybean yield at ripening stage. A previous study showed that intercropping increased N concentration in junction crops [41], but this was not observed after maize tasseling stage in our study. Thus, the increase in total N accumulation of crops in intercropping systems was mainly because intercropping promoted biomass production. Our finding that accumulation of N by crops in intercropping systems was higher than that in maize monoculture was consistent with the conclusion of Li et al. [20], but it was contradict with Zhang et al. [42] who observed a higher amount of N accumulation in monoculture than intercropping.

An optimal cropping system should aim at having a positive residual effect and increasing or at least not reducing yield of a subsequent crop. A promoted yield production of a subsequent crop by intercropping systems was observed in some studies [43,44], but not in others [45]. In the present study, yields of wheat following 4M:6S was the highest among all the treatments and its soil (0–100 cm) nitrate-N accumulation was significantly lower than that following maize monoculture. This provides additional proof of advantage existing in 4M:6S intercropping.

## Conclusions

Compared to conventional monoculture of maize, both maize ǁ soybean and maize ǁ red bean had significant advantage in yield, economy, land utilization ratio and reducing soil nitrate-N accumulation, as well as better residual effect on the subsequent wheat crop. In particular, maize ǁ soybean performed best, and was thus identified as the optimal summer cropping system in this study. Intercropping systems could reduce N fertilizer use and increase relative biomass of intercropped maize, as a result of high photosynthetic efficiency of border rows and sufficient nitrate supply during symbiotic period. Noticeably, intercropping advantage was not inherent but began to emerge at tasseling stage after N topdressing for maize. 4M:6S was the best intercropping system in this study, as it had the largest LER, crop total N accumulation and economic benefit. In addition, compared to maize monoculture, 4M:6S significantly reduced nitrate-N accumulation in the soil after harvest of both summer crops and winter wheat, and it even slightly increased wheat yield. The most important advantage of 4M:6S was to increase biomass of intercropped maize and soybean, which further led to the increase of total N accumulation by crops as well as economic benefit. In conclusion, alternating four maize rows with six soybean rows is the optimum row ratio in maize || soybean system, though this needs to be further confirmed by pluri-annual trials.
